# Dual Anta-Inhibitors Targeting Protein Kinase CK1δ and A_2A_ Adenosine Receptor Useful in Neurodegenerative Disorders

**DOI:** 10.3390/molecules28124762

**Published:** 2023-06-14

**Authors:** Beatrice Francucci, Simone Angeloni, Diego Dal Ben, Catia Lambertucci, Massimo Ricciutelli, Andrea Spinaci, Aleksei Smirnov, Rosaria Volpini, Michela Buccioni, Gabriella Marucci

**Affiliations:** Medicinal Chemistry Unit, School of Pharmacy, University of Camerino, 62032 Camerino, Italy; beatrice.francucci@unicam.it (B.F.); simone.angeloni@unicam.it (S.A.); diego.dalben@unicam.it (D.D.B.); catia.lambertucci@unicam.it (C.L.); massimo.ricciutelli@unicam.it (M.R.); andrea.spinaci@unicam.it (A.S.); rosaria.volpini@unicam.it (R.V.); gabriella.marucci@unicam.it (G.M.)

**Keywords:** A_2A_AR antagonists, CK1δ inhibitors, A_2A_/CK1δ dual inhibitors, neuroinflammation, cytokine, neuroprotection

## Abstract

Currently, the number of patients with neurodegenerative pathologies is estimated at over one million, with consequences also on the economic level. Several factors contribute to their development, including overexpression of A_2A_ adenosine receptors (A_2A_AR) in microglial cells and up-regulation and post-translational alterations of some casein kinases (CK), among them, CK-1δ. The aim of the work was to study the activity of A_2A_AR and CK1δ in neurodegeneration using in-house synthesized A_2A_/CK1δ dual anta-inhibitors and to evaluate their intestinal absorption. Experiments were performed on N13 microglial cells, which were treated with a proinflammatory CK cocktail to simulate an inflammatory state typical of neurodegenerative diseases. Results showed that the dual anta-inhibitors have the ability to counteract the inflammatory state, even if compound **2** is more active than compound **1**. In addition, compound **2** displayed an important antioxidant effect similar to the reference compound ZM241385. Since many known kinase inhibitors are very often unable to cross lipid bilayer membranes, the ability of A_2A_/CK1δ double anta-inhibitors to cross the intestinal barrier was investigated by an everted gut sac assay. HPLC analysis revealed that both compounds are able to cross the intestinal barrier, making them promising candidates for oral therapy.

## 1. Introduction

The etiopathogenetic mechanisms of neurodegenerative pathologies such as amyotrophic lateral sclerosis (ALS), Parkinson’s disease (PD), and Alzheimer’s disease (AD) are not yet fully understood. However, they are certainly multifactorial pathologies in which different factors contribute to their development. Nevertheless, it is well known that neurodegenerative diseases are associated with neuroinflammation, and the key regulators of inflammatory responses in the central nervous system (SCN) are microglia and astrocytes.

Several studies highlighted up-regulation and post-translational alterations of casein kinases (CK), especially CK-1δ, in various forms of cancer and neurodegenerative diseases. CK-1δ phosphorylates more than 150 proteins in vitro, regulating several physiological and pathological processes. It participates in the phosphorylation of the same proteins that contribute to the neurodegenerative process in various diseases, such as alpha-synuclein (α-Syn), TAU, PARKIN, and TDP-43 (TAR-DNA binding protein-43) [1,2,3].

α-Syn is a presynaptic protein that is the major constituent protein of Lewy bodies, the hallmark of Parkinson’s disease (PD) [4,5]. Accumulations of α-syn were found in the brain of patients with AD [6,7,8,9], as well as in patients with synucleinopathies, such as PD, dementia with Lewy bodies (DLB) [10,11], and multiple system atrophy [12,13]. Aggregates of α-Syn were detected colocalized with CK-1δ in the autoptic brain of patients affected by AD or synucleinopathies, suggesting a close relationship between CK-1δ and neurodegeneration [12].

Tau is another protein that represents the hallmark of multiple neurodegenerative diseases. Its aberrant phosphorylation by CK1δ converts its soluble form into paired helical filaments (PHF), leading to the development of neurofibrillary tangles (NFTs) that cause the progression of neurodegenerative disorders, including AD and other tauopathies [14].

PARKIN is another CK-1δ target protein necessary for the survival of neurons whose phosphorylation induces a reduction in solubility, leading to its aggregation and consequent inactivation. Therefore, the regulation of PARKIN phosphorylation status through CK-1δ inhibition could be a strategy to reduce PARKIN inactivation and represent a new therapeutic approach in the treatment of PD [15].

CK-1δ also causes the pathological phosphorylation of the TDP-43 protein, which results in upregulated in spinal cord tissue in ALS and other neural disorders. It is known that overexpression of this protein produces neuronal death in different animal models due to its toxic effect on proteins. Experiments performed in drosophila models highlighted that CK-1δ inhibition precludes TDP-43 phosphorylation in vitro, reducing its neurotoxicity [16,17,18]. These data were confirmed in vivo, in transgenic mice, using the CK-1δ inhibitor IGS-2.7. Experiments revealed the importance of modulating TDP-43 toxicity by CK-1δ inhibition in spinal motor neuron preservation [19].

Despite the many advances accomplished in recent years, the challenge against neurodegenerative diseases is still open, and given the complexity of the field, researchers have been recently focusing on the discovery of dual molecules able to interact with multiple targets simultaneously.

Recent evidence shows the involvement of the A_2A_ adenosine receptor (A_2A_AR) in the neuroinflammation that accompanies neurodegenerative diseases [20,21,22,23,24]. A_2A_AR is highly expressed in microglial cells, a type of glial cell that is responsible for the first and main active immune defense in the central nervous system (CNS) [25]. During a brain insult, A_2A_AR expression is augmented, leading to pathological signal transductions such as increased proinflammatory cytokine release. A_2A_AR antagonists are able to restore the normal functions of microglial processes, underlining the potential of these ligands in the pharmacological therapy of neurodegenerative diseases [26,27,28,29,30]. New therapeutic agents endowed with synergistic effects able to inhibit the enzyme isoform CK1δ and antagonize A_2A_AR could, therefore, represent a suitable strategy for the treatment of chronic neurodegenerative diseases. The aim of the work was, therefore, to evaluate the effects of dual A_2A_/CK1δ anta-inhibitors, synthesized in-house and recently published, in neuroinflammation and their absorption across the intestinal barrier. The experiments were performed in microglial cells, where the neuroinflammation was caused by a cocktail of proinflammatory cytokines and in everted gut sacs.

## 2. Results and Discussion

**Figure 1:**
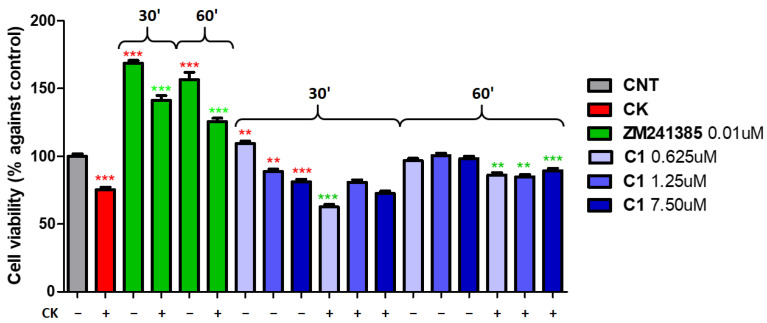
Effect of compound **1** in comparison with reference compound ZM241385. Percentage of cell viability after treatment with ligands alone or in the presence of CK cocktail. Results are expressed as the mean of 3–5 independent experiments. ** *p* < 0.01, *** *p* < 0.001 of treated cells against control. Red asterisks refer to the control, while green ones refer to the CK cocktail.

Neurodegeneration is usually associated with neuroinflammation, which is an innate immune response of brain cells to block infection and eliminate toxins, pathogens, cell debris, etc. The initial role of this mechanism is protective, but the prolonged effect induces major neurodegenerative and psychiatric disorders. In fact, the main factors that lead to inflammation are cytokines (CKs), chemokines, reactive oxygen species, and secondary messengers. Change in microglial morphology characterizes the most common neuropathologies in nearly all CNS diseases. Microglial cells express Ars, through which adenosine acts as an immune regulation mediator. Thus, it is possible to utilize selective agonists, antagonists, and allosteric modulators to regulate the activity of these receptors, leading to desired therapeutic effects. Additionally, there is an enhancement of cytokines in proinflammatory response [31,32]. In a recent paper, a set of di- and tri-substituted adenine derivatives was obtained and studied for their capability to inhibit the CK1δ enzyme and to antagonize adenosine receptors (ARs) [33]. Some of these compounds acted as dual anta-inhibitors, demonstrating a suitable CK1δ inhibitory activity combined with a high binding affinity, especially for the A_2A_AR. Among them, 9-Cyclopentyl-N2,N2-dimethyl-9H-purine-2,6-diamine (**1**) and *N^6^*-methyl-(2-benzimidazolyl)-2-dimethyamino-9-cyclopentyladenine (**2**) (Figure 1) showed the best balance of A_2A_R affinity and CK1δ inhibitory activity, while 2-((2-Chloro-9-cyclopentyl-9H-purin-6-yl)amino)acetonitrile (**3**) and 2-chloro-9-cyclopentyl-8-(furan-2-yl)-9H-purin-6-amine (**4**) (Table 1) were more active versus A_2A_R and CK1δ, respectively.

The results of the binding assay and enzymatic inhibition activity test are reported in Table 1A,B, respectively, in comparison with the reference compounds (PF-670462 for CK1δ and ZM241385 for A_2A_AR).


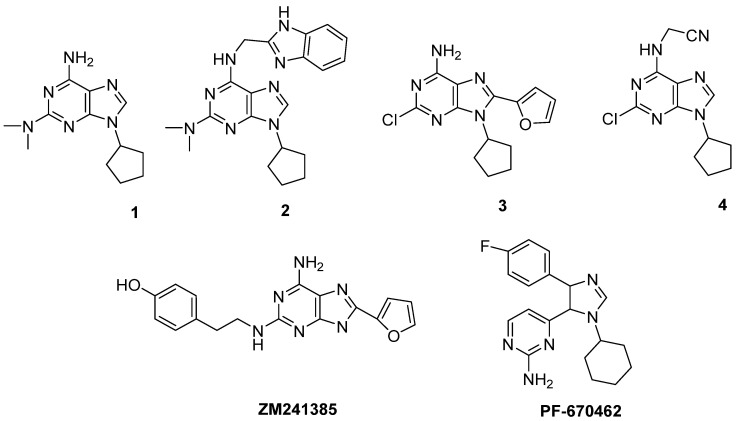


Starting from these observations, since this double effect could be a suitable strategy for the treatment of chronic neurodegenerative diseases, in which neuroinflammation plays a crucial role, these compounds were tested on an in vitro model of neuroinflammation in comparison with the reference compounds. The experiments were performed using N13 microglial cells. The colorimetric method CellTiter 96^®^ AQueous One Solution Cell Proliferation assay was used to determine the number of live cells in cytotoxicity or proliferation assays. The tetrazole compound MTS was bio-reduced by the cells into formazan, a colored product soluble in the cellular medium. The percentage of cell viability was analyzed through an absorbance reading at 492 nm. The concentrations of ligands were chosen based on the results of binding assays. Specifically, lower, similar, and higher concentrations than the K_i_ value were used. The concentrations tested for compounds **1** and **2** were 0.625, 1.25, and 7.5 µM and 0.375, 0.75, and 4.5 µM, respectively, while the referent compound ZM241385 was used at 0.01 µM, the best concentration determined in the previous work, at 30 and 60 min of incubation in the presence and in the absence of a proinflammatory cocktail of cytokines (TNF-α, 20 ng/mL; IL-1β, 20 ng/mL; IFN-γ 20 ng/mL) (CK) (Figure 1) [27].

**Figure 2:**
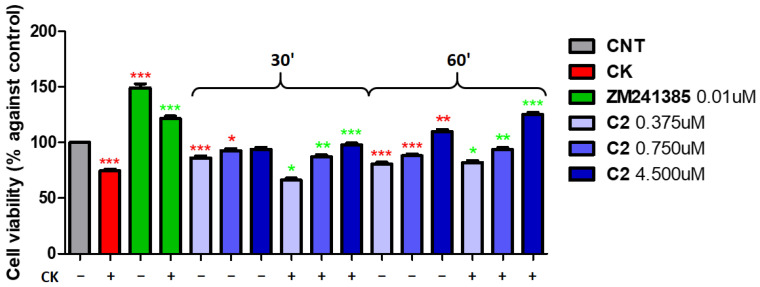
Effect of compound **2** in comparison with reference compound ZM241385. Percentage of cell viability after treatment with ligands alone or in the presence of CK cocktail. Results are expressed as the mean of 3−5 independent experiments. * *p* < 0.05, ** *p* < 0.01, *** *p* < 0.001 of treated cells against control. Red asterisks refer to the control, while green ones refer to the CK cocktail.

Since ZM241385 has comparable activity at 30 and 60 min, we chose to use this compound as a reference at 60 min of incubation in order to standardize all experiments. The results are shown in Figure 2. At 30 min of compound **1** incubation in the absence of CK cocktail, only the lowest concentration tested, 0.625 µM, produced a significant increase in cell viability (112% ± 2.4 vs. control) while being inferior to the reference compound ZM241385 at 0.01 µM (155% ± 1.9 vs. control). In addition, the highest concentration of compound **1**, 7.5 µM, produced a lower cell viability with respect to the control, suggesting a tendency to toxicity at 30 min but not at 60 min since the same concentration showed a cell viability similar to the control. As for compound **2** at 30 min, all concentrations produced a decrease in cell viability, particularly significant at the concentration of 0.375 µM (86% ± 1.2 vs. control) (Figure 2).

**Figure 3:**
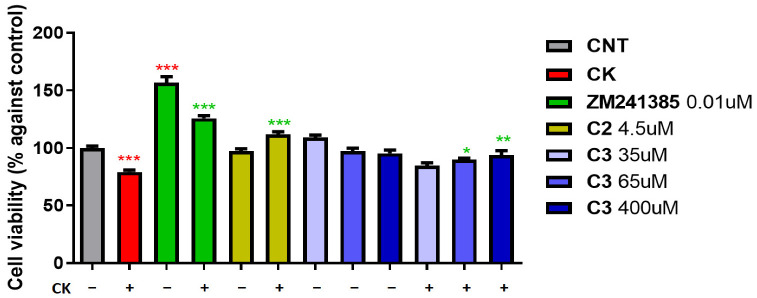
Effects of compounds **2** and **3** in comparison with reference compound ZM241385. Percentage of cell viability after treatment with ligands alone in the absence or in the presence of CK cocktail. Results are expressed as the mean of 3−5 independent experiments. * *p* < 0.05, ** *p* < 0.01, *** *p* < 0.001 of treated cells against control. Red asterisks refer to the control, while green ones refer to the CK cocktail. Red asterisks refer to the control, while green ones refer to the CK cocktail.

On the other hand, at 60 min of incubation at 4.5 µM, there was a significant increase in cell viability (109% ± 1.4 vs. control), while concentrations 0.375 and 0.75 µM produced a decrease in cell viability similar to 30 min of incubation. As expected, the incubation of cells with the CK cocktail caused a significant decrease in vitality (74% ± 0.3 vs. control). Given the nature of compounds, dual A_2A_/CK1δ anta-inhibitors, their neuroprotective effect against a neuroinflammatory insult was investigated following two different approaches aimed at studying the antagonist activity on the A_2A_AR and the inhibitor effect on CK1δ. In the experiment to study the A_2A_AR antagonist effect, microglial cells were pretreated with 20 ng/mL of CK cocktail for 48 h, and subsequently, compounds **1** and **2** at 0.625, 1.25, and 7.5 µM and 0.375, 0.75, and 4.5 µM, respectively, for 30 and 60 min or the referent compound ZM241385 at 0.01 µM were added. The results showed that both compounds **1** and **2** were unable to rescue the inflammatory damage at lower concentrations at 0.625 and 0.375 µM, respectively, at 30 min of incubation, while the other concentrations showed a suitable ability to rescue the effect induced by the CK cocktail at both 30 and 60 min of incubation. It is important to note that the lower concentrations of both compounds after 60 min of incubation were able to counteract the effect of the CK cocktail, even if the best concentrations resulted in 7.5 µM for compound **1** and 4.5 µM for compound **2**. These concentrations produced an increase in cell vitality, reaching a viability of 89% ± 2.4 and 125% ± 3.1 with respect to cell viability in the presence of cytokine (74% ± 0.3 vs. control), demonstrating that these compounds can counteract the damage produced by the CK cocktail. Compound **2** at 4.5 µM after 60 min of incubation showed a similar effect on referent compound ZM241385. These results underline that the best incubation time was 60 min, and for this reason, further experiments were carried out at 60 min of incubation. Compound **3**, which was inactive as a CK1δ inhibitor, was studied as an A_2A_AR antagonist in comparison with compound **2** at 4.5 µM, a concentration that produced major activity. The concentrations used were 35, 65, and 400 µM at 60 min of incubation. The results showed that compound **3** is also able to counteract the effect of the CK cocktail but to a lesser extent than compound **2**. In fact, compound **2** at 4.5 µM produced a cell vitality increase of up to 112% ± 3.1, while compound **3** at 400 µM caused an increase of up to 93 ± 1.1 (Figure 3).

**Figure 4:**
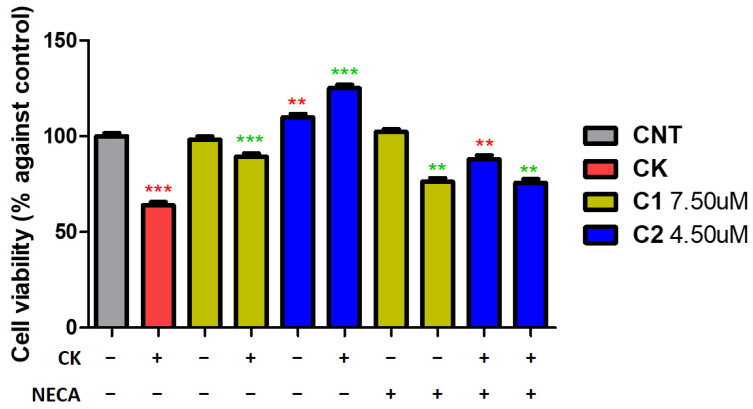
Effects of compounds **1** and **2**. Percentage of cell viability after treatment with ligands alone or co-administered with NECA in the absence or in the presence of CK cocktail. Results are expressed as the mean of 3–5 independent experiments. ** *p* < 0.01, *** *p* < 0.001 of treated cells against control. Red asterisks refer to the control, while green ones refer to the CK cocktail.

To verify that this block of neuroinflammation was inducible in A_2A_AR antagonism, the dual A_2A_/CK1δ anta-inhibitors, compounds **1** and **2**, were analyzed in the presence of the non-selective agonist of adenosine receptors (2S,3S,4R,5R)-5-(6-amino-9H-purin-9-yl)-N-ethyl-3,4-dihydroxytetrahydrofuran-2-carboxamide (NECA) (Figure 4). Since compound **3** is an A_2A_AR antagonist and is not active at CK1δ, it was not included in the experiment.

The experiments were carried out by pretreating cells with 20 ng/mL of CK cocktail for 48 h, then adding them to compounds **1** and **2** at doses more efficacious, 7.5 µM and 4.5 µM, respectively, for 60 min, and the NECA at 10 µM. As expected, findings obtained showed that the effect after CK cocktail damage of compounds **1** and **2** in the presence of NECA is significantly lower than that obtained in the absence of NECA. This demonstrates that the effect is due to A_2A_AR interaction since agonist NECA is able to displace compounds **1** and **2** from A_2A_AR, generating a decrease in compounds to counteract the neurogenerative effect of CK.

Since the overexpression and aberrant activity of CK1δ were connected to hyperphosphorylation of key proteins implicated in the development of neurodegenerative disorders, to study the effect of CK1δ inhibition in neuroprotection, experiments were performed by pretreating the neuronal cells with compounds under study for 60 min of incubation before adding the 20 ng/mL of CK cocktail for 48 h.

**Figure 5:**
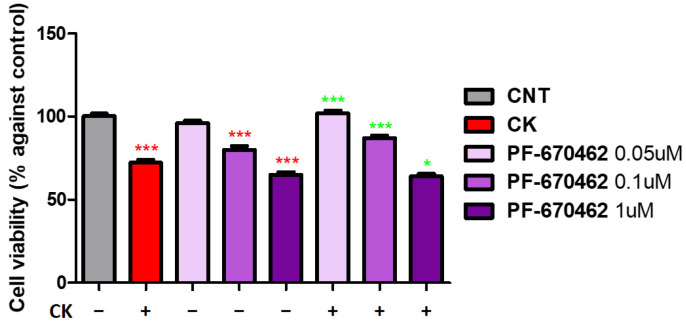
Effect of reference compound PF-670462. Percentage of cell viability after treatment with ligand alone or in the presence of CK cocktail. Results are expressed as the mean of 3–5 independent experiments. * *p* < 0.05, *** *p* < 0.001 of treated cells against control. Red asterisks refer to the control, while green ones refer to the CK cocktail.

Initially, we studied the effect of a known CK1δ inhibitor, PF-670462, at 0.05, 0.1, and 1 µM according to the IC_50_ values calculated in the enzymatic inhibition experiments (Table 1B) for 60 min of incubation alone and before adding the CK cocktail for 48 h (Figure 5).

The results suggest that PF-670462 alone does not affect cell viability at 0.05, but at 0.1 and 1 µM, it decreases cell viability up to 75% ± 1.4 and 60% ± 2.9, respectively. This phenomenon could be due to the inhibition of protein kinases and phosphatases that regulates many processes in living cells through modification of serine, threonine, and tyrosine residues that influence DNA replication, transcription, and translation, leading to decreased cell growth [34,35,36,37]. This situation changed when there was the insult of CK cocktail; in fact, the concentrations of 0.05 and 0.1 µM produced a significant neuroprotective effect with an increase in cell vitality up to 96% ± 3.1 and 78% ± 3.5, respectively, with respect the cell viability in the presence of cytokine (69% ± 0.3 vs. control). The concentration of 1 µM produced no significant decrease in cell viability.

The same experiments were performed with compounds **1**, **2**, and **4** in comparison with PF-670462. These experiments were not executed with compound **3** since this compound is not active as an enzymatic CK1δ inhibitor. The concentrations utilized were 40, 80, and 820 µM for compound **1** and 25, 50, and 500 µM for compounds **2** and **4** according to the IC_50_ values calculated in the enzymatic inhibition experiments (Table 1B).

**Figure 6:**
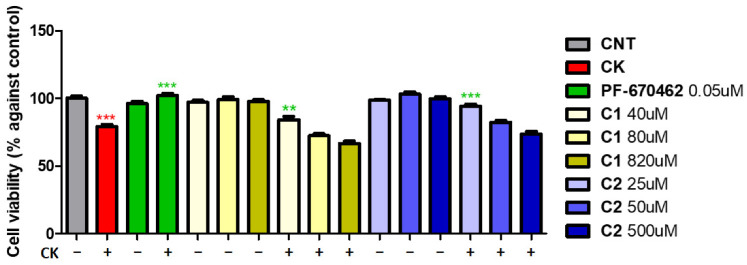
Effects of compounds **1** and **2** in comparison with reference compound PF-670462. Percentage of cell viability after treatment with ligands alone or in the presence of CK cocktail. Results are expressed as the mean of 3–5 independent experiments. ** *p* < 0.01, *** *p* < 0.001 of treated cells against control. Red asterisks refer to the control, while green ones refer to the CK cocktail.

Results shown in Figure 6 demonstrate that compound **1**, which exhibits greater A_2A_AR antagonism than enzymatic inhibition, is less effective in neuroprotection with respect to compound **2**, which exhibits a suitable enzymatic inhibition. The concentration that produces the major protective effect of compound **1** is 40 µM with an increase in cell viability of up to 85% ± 1.9. In contrast, the lower concentrations of compound **2** (25 µM) produce an increase in cell viability of up to 94% ± 2.2 with respect to the cell viability in the presence of cytokine (79% ± 1.1 vs. control), demonstrating a suitable neuroprotective agent. A possible explanation of this activity is that in many neurodegenerative disorders, hyper-phosphorylation is observed. Since phosphorylation by CK1δ controls many protein activities and their stability, localization, and interaction with other proteins, this post-translational modification is capable of changing protein functions by either allosteric interaction or binding to regulatory domains [38,39].

It has an important impact on processes such as DNA replication, transcription and translation, cell metabolism, apoptosis, and stress and immunological response [36]. Therefore, when the CK1δ inhibitor was added before, the CK insult inhibited the hyperphosphorylation-producing cell proliferation by reducing the proinflammatory factors effect.

**Figure 7:**
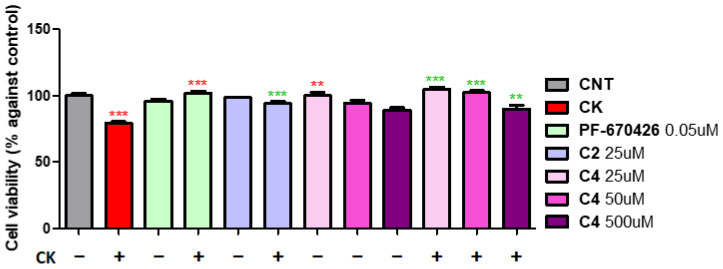
Effects of compounds **2** and **4** in comparison with reference compound PF-670462. Percentage of cell viability after treatment with ligand alone or in the presence of CK cocktail. Results are expressed as the mean of 3–5 independent experiments. ** *p* < 0.01, *** *p* < 0.001 of treated cells against control. Red asterisks refer to the control, while green ones refer to the CK cocktail.

Compound **4**, which was inactive as an A_2A_AR antagonist, was studied as a neuroprotective agent in comparison with compound **2** at 25 µM, a concentration that produced a major neuroprotective effect. The concentrations used were 25, 50, and 500 µM at 60 min of incubation. Results showed that compound **4** is able to restore the effect of the CK cocktail (78% 1.3 vs. control) at all concentrations used better than compound **2**. The concentration that produced the highest increase in cell viability was 25 µM (108% ± 0.5) even if 50 and 500 µM also showed suitable activity (102%± 1.0 and 90% ± 1.1) (Figure 7).

**Figure 8:**
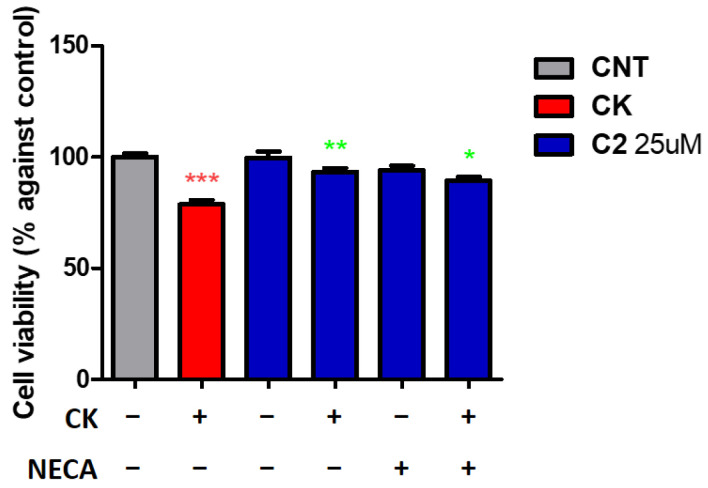
Effect of compound **2**. Percentage of cell viability after treatment with ligand alone or co-administered with NECA in the absence or in the presence of CK cocktail. Results are expressed as the mean of 3–5 independent experiments. * *p* < 0.05, ** *p* < 0.01, *** *p* < 0.001 of treated cells against control. Red asterisks refer to the control, while green ones refer to the CK cocktail.

The last experiment was carried out to understand if the activity of dual A_2A_/CK1δ anta-inhibitors in the second experiment was due only to CK1δ inhibition. The experiment was performed with compound **2** in the presence of NECA. In particular, compound **2** at 25 µM was incubated for 60 min before adding the CK cocktail for 48 h, and then the NECA was added at 10 µM. NECA, in this case, was ineffective in counteracting the effect of compound **2**, demonstrating that the result obtained is due only to CK1δ inhibition (Figure 8).

To prove the validity of the results obtained, the most significant experiments were also performed to quantify the ATP, which is an indicator of metabolically active cells, and its amount is proportional to the number of viable cells in culture (CellTiter-Glo^®^ Luminescent Cell Viability Assay). Results obtained were not significantly different from those previously found and are not reported.

**Figure 9:**
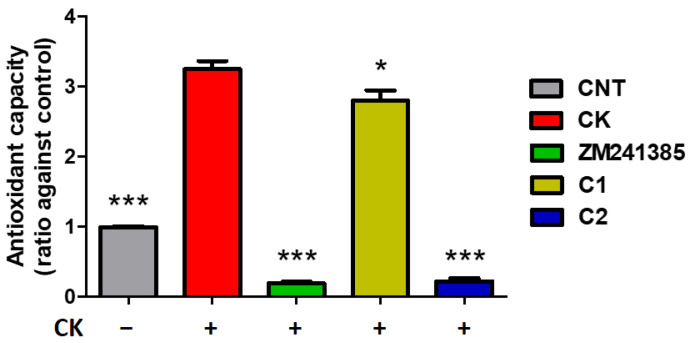
Antioxidant effects of ZM241385 and compounds **1** and **2** after pretreatment with CK cocktail. Ratio of NO_2_− production after treatments. Results are expressed as the mean of 3–5 independent experiments. * *p* < 0.05, *** *p* < 0.001 of treated cells against CK.

In addition, since oxidative stress has received extensive attention as one important entry point in the pathogenesis of neurodegenerative diseases, the potential antioxidant profile of compounds **1** and **2** was investigated. Oxidative stress and neuroinflammation play synergistic roles in the promotion of neurodegenerative disease progression [40,41]. During a CNS insult, there is an activation of microglial cells, which stimulate immune responses to instigate tissue repair. If the triggering cause of the immune response is eradicated, the immune response resolves, but if the initial stress persists, it will lead to an overproduction of neurotoxic factors such as chemokines and proinflammatory cytokines such as Tumor Necrosis Factor-α (TNF-α) and Interleukin-1β (IL-1β). This pathological state will lead to an overproduction of ROS and a state of oxidative stress. High levels of ROS inactivate or damage proteins, leading to cellular degeneration and death. Furthermore, ROS can activate proinflammatory pathways, further promoting a harmful environment for vulnerable neurons. In light of this, the potential antioxidant capacity of compounds was studied. Compounds **1** and **2** were evaluated by Griess Reagent System, utilizing the concentrations found to be most active in previous assays (compound **1**: 7500 nM; compound **2**: 4500 nM). Compound **1** and compound **2** were incubated for 1 hr in comparison with the reference compound and ZM241385 (10 nM). Compound **1** showed no significant antioxidant activity, while compound **2** displayed an important antioxidant effect similar to that of the reference compound. The amount of nitrite produced after compound **2** treatment was inferior to that revealed in the control, demonstrating that this compound possesses a robust antioxidant effect. The oxidative stress caused by the CK cocktail was successfully compensated by both compound **2** and the reference compound (Figure 9). The combination of anti-inflammatory and antioxidant activities makes compound **2** particularly appealing in counteracting two of the factors involved in the pathogenesis of neurodegenerative pathologies.

Despite these remarkable assumptions, as many known kinase inhibitors are very often unable to cross lipid bilayer membranes, including the blood–brain barrier, and are, therefore, only suitable for non-central nervous system disorders, the ability of A_2A_/CK1δ double anta-inhibitors to cross the intestinal barrier was investigated. The experiment was performed using the everted gut sac model. The everted sac model is an efficient tool for studying in vitro drug absorption mechanisms, intestinal metabolism of drugs, the role of transporters in drug absorption, and investigating the role of intestinal enzymes during drug transport through the intestine. Compounds **1** and **2** were measured by liquid chromatography-mass spectrometry (LC-MS) in the mucosal and serosal media to provide a complete picture of the transport.

**Figure 10:**
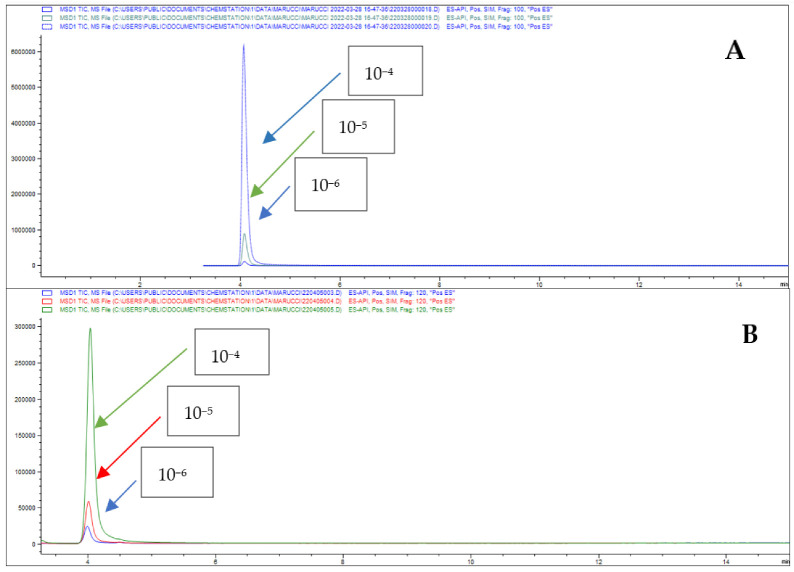
Chromatograms of compound **1** (panel **A**) and **2** (panel **B**) standard solutions.

The analytical methods used for compound quantitation were validated by studying linearity, limit of detection (LOD), limit of quantification (LOQ), and precision. Linearity was tested by injecting different concentrations of compounds **1** and **2**, and a calibration curve with a determination coefficient (R^2^) for each analyte was plotted and measured. R^2^ was 0.9987 and 0.9991 for compound **1** and compound **2**, respectively. For compound **1**, LOQ and LOD were 0.1 μM and 0.033 μM, while for compound **2**, they were 0.125 μM and 0.0417 μM. The method showed satisfactory precision since the intra- and inter-day repeatability for both compounds ranged from 2.1 to 6.0%. The chromatograms acquired in SIM mode of compound **1** and compound **2**, studied as standard solutions at three different concentrations, are reported in Figure 10.

**Figure 11:**
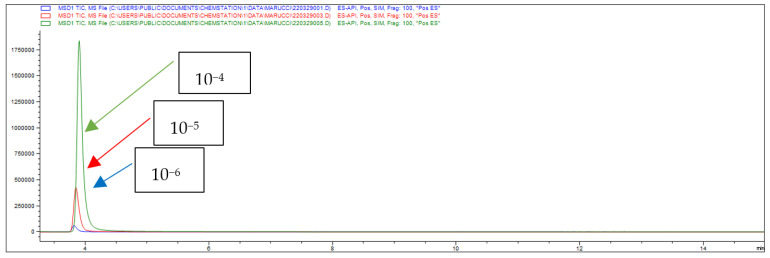
Chromatograms of compound **1** absorbed in the duodenum.

Compounds **1** and **2** were incubated in the duodenum for 60 min at concentrations of 10^−4^, 10^−5^, and 10^−6^ M to evaluate their absorption. The duodenum was chosen because, in a precedent paper, it was demonstrated that it is the best part of the rat intestine to evaluate drug absorption [42]. The chromatogram obtained of compound **1** after absorption is reported in Figure 11.

Results show that it is well absorbed at 10^−4^ M and 10^−5^ M, while the absorption at 10^−6^ M is lower than the other two concentrations.

**Figure 12:**
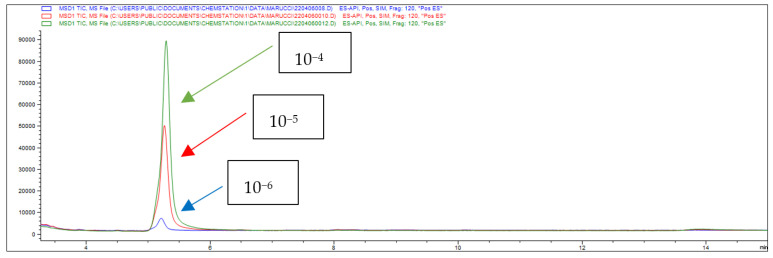
Chromatograms of compound **2** absorbed in the duodenum.

The same situation is observable on the chromatogram of compound **2** (Figure 12).

Also in this case, the chromatogram overlaps with the one shown in Figure 10B, but unfortunately, compound **2** was well absorbed at 10^−4^ M and 10^−5^ M, while the absorption at 10^−6^ M was not well detected.

In order to evaluate the absorption of compounds **1** and **2**, the permeability coefficient (P*_app_*), drug absorption (*A*%), and drug retention (*Ad*%) were determined. The values obtained are described in Table 2 and Table 3. The concentrations 10^−4^, 10^−5^, and 10^−6^ M correspond to 24.6, 2.46, and 0.246 μg/mL of compound **1** and to 37.6, 3.76, and 0.376 μg/mL of compound **2** due to different molecular weights. Glucose was used to verify the integrity and viability of the gut sacs. Intestine sacs were exposed to the concentrations of compounds under study with respect to glucose-treated tissues. After incubation time, fluids from the mucosal (outside the sacs) and serosal (inside the sacs) sides of the rat gut sacs were collected and detected by HPLC-MS analysis. As expected, the amount of the absorbed analytes was concentration dependent even if compound **1** was much better absorbed with respect to compound **2** (Table 2 and Table 3). In fact, the lower concentration tested of compound **2** (10^−6^ M) was not detectable by HPLC analysis. Data are reported as means ± SE (*n* = 4–5 experiments). The amount of compounds absorbed compared to the initial concentration is statistically different (*p* < 0.05).

Data obtained from apparent permeability studies further confirmed the better absorption of compound **1** with respect to compound **2**, underlined by the highest permeability coefficient (P*_app_*) value (33 × 10^−6^ vs. 16.1 × 10^−6^ cm/s at 10^−4^ M, Table 4 and Table 5). The high values of P*_app_* obtained with both analytes confirm the participation of an active absorption mechanism.

The percentages of absorbed (*A*%) and retained (*Ad*%) analytes on intestinal tissue showed that ligand **1** was supposedly much more absorbed, displaying an *A*% of 47% (Table 4). The amount of retained analyte by the everted gut sac tissues ranged from 2 to 35%, increasing the concentration from 10^−4^ to 10^−6^ M, respectively. The profile of compound **2** was apparently quite different since data obtained show that it was much less absorbed (23 and 15% at 10^−4^ and 10^−5^, respectively) and much more retained (55 and 66% at 10^−4^ and 10^−5^, respectively) (Table 5). A careful HPLC analysis revealed that the compound was not recovered as it was partially converted into its metabolite, compound **1**. Results obtained indicate that both compounds represent promising candidates for oral administration in a therapy aimed at relieving the inflammation accompanying neurodegenerative diseases.

The data shown in Table 4 and Table 5 represent the means ± SE (*n* = 4–5 experiments). The amount of compound percentage values is statistically different (*p* < 0.05).

## 3. Materials and Methods

### 3.1. Test Materials

Compounds **1**–**4** and NECA were designed and synthesized at the School of Pharmacy of Camerino University (Italy) and dissolved as reported before [28]. Briefly, the 10 mM stock solutions were prepared in dimethylsulfoxide (DMSO) and then diluted with water to reach the desired concentration. In any case, the DMSO concentration in each well was equal to or lower than 0.5% and did not affect cell viability. PF 670462 and TC199 medium (with Hank’s salts and L-glutamine, without NaHCO_3_) were purchased from Sigma-Aldrich (Milan, Italy). All material used for cell maintenance was bought from EuroClone S.p.A. (Milan, Italy), and CellTiter 96^®^ Aqueous One Solution Cell Proliferation and CellTiter-Glo^®^ Luminescent Cell Viability Assays were obtained from Promega (Milan, Italy).

### 3.2. Animals

Ten adult male Wistar rats (250–300 g; 11–12 weeks) were purchased from Charles River Laboratories Srl (Lecco, Italy). All animals were maintained under controlled conditions, including a 12:12 h light/dark cycle (lights off at 8.00 a.m.) and constant temperature (20–22 °C) and humidity (45–55%). The day before the experiment, the test animals were fasted. Animal experiments were performed according to the European Communities Council Directive of 24 November 1986 (86/609/EEC) [43].

### 3.3. Cells Culture

N13 cells were grown in DMEM High Glucose enriched with 10% fetal bovine serum, 100 U/mL penicillin, 100 µg/mL streptomycin, 1mM sodium pyruvate, and 2 mM L-glutamine in a humidified atmosphere with 5% CO_2_ at 37 °C.

### 3.4. Cell Titer 96^®^ Aqueous One Solution Cell Proliferation Assay

**Scheme 1:**
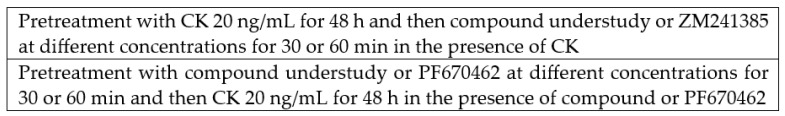
Drug application protocol.

N13 cells were plated in 96-well plates at the confluence of 1 × 10^4^ cells/well and incubated overnight. Cells were divided into two groups in order to study the activity of compounds on A_2A_AR and CK1δ (Scheme 1). The following day, cells of the first group were pretreated with a proinflammatory cocktail of cytokines (TNF-α, 20 ng/mL; IL-1β, 20 ng/mL; IFN-γ 20 ng/mL) for 48 h, and then we added 2 μL of ligand, or reference compound ZM241385, to each well for 30 or 60 min. Cells of the second group were pretreated with 2 µL at different concentrations of understudy ligand, or the reference compound PF670462, for 30 or 60 min and then with further addition of a proinflammatory cocktail of cytokines for 48 h (Scheme 1). After treatment, 20 µL of CellTiter 96^®^ AQueous One Solution Reagent was added to each well to a final volume of 100 µL. After 1 h of incubation, absorbance was measured at 492 nm in the microplate reader GENiosPro. Cell viability was estimated as the relation between the OD of treated cells and the OD of untreated cells in percentage. An untreated control and a control with the solvent were run. Experiments were performed in triplicate.

### 3.5. CellTiter-Glo^®^ Luminescent Cell Viability Assay

N13 cells were plated in 96-well plates at the confluence of 1 × 10^4^ cells/well and incubated overnight. The day after, an addition of 2 μL of ligand or CK cocktail was performed to the wells. After treatment, cells were lysed through incubation for 30 min with the CellTiter-Glo reagent. The determination of viable cells in culture was carried out by measuring, through the microplate reader GENiosPro, the luminescence signal caused by the presence of ATP on viable cells. The results are expressed as the percentage of control cells that were not treated. An untreated control and a control with the solvent were run. All experiments were performed in triplicate.

### 3.6. Griess Assay

The N13 cell line was introduced into a 96-well UV-VIS transparent plate at a population of 1 × 10^4^ cells per well in 98 μL of DMEM and incubated overnight. The day after, 2 µL volume of substance (compound **1**, 7500 nM or compound **2**, 4500 nM or reference compound ZM241385, 10 nM) or CK cocktail were added to the wells. After 24 h of incubation, the media from each well was transferred into another 96-well plate, and then 50 µL of the sulphanilamide solution was placed into the wells. After 10 min of incubation at room temperature (rt), 50 µL of the NED solution was dispensed to all wells, and the plate was incubated at rt for an additional 5–10 min. After half an hour, absorbance was measured in the microplate reader GENiosPro using a 520–550 nm filter. All measurements were reproduced in 3–5 replicates [27]. The nitric oxide formation was investigated by measuring nitrite (NO^2−^), which is one of two primary breakdown products of NO. A nitrite standard reference curve was used for quantitative evaluation of the concentration of NO^2−^ in samples.

### 3.7. Everted Gut Sac Studies

Adult male Wistar rats (250–300 g; 11–12 weeks) were sacrificed by cervical dislocation, the small intestine was removed, and the duodenum segment was isolated and washed in TC199 medium. The intestine was divided into segments of 2–2.5 cm in length (4 for each animal), and each of them was everted on a glass rod of 2.5 cm in diameter. All segments were closed at one end with a silk thread, filled with fresh oxygenated TC199 medium, and closed at the other end, creating a shut section (sac). Each sac was placed in a 50 mL beaker containing 9.0 mL of medium aerated with oxygen (O_2_/CO_2_, 95%:5%) and thermostated at 37 °C. A total of 1 mL of medium containing one of the samples at a concentration of 1 uM, 10 uM, or 100 uM was added. Each concentration was tested in duplicate in three different animals. In order to mimic the intestine peristalsis, gut sacs were incubated in an oscillating water bath (60 cycles/min) [42,44]. After 1h incubation, sacs were removed, washed in saline solution (0.9% NaCl), and wiped with filter paper. The content of each sac (serosal fluid) was collected into a 1.5 mL Eppendorf tube, and the sac was weighed before and after serosal fluid removal to calculate the exact volume contained inside. Samples were centrifuged for 15 min. at 13,500× *g*, and the supernatant aqueous solution was filtered through a microporous membrane (0.45 μm) for high-performance liquid chromatography (HPLC). The amount of compound **1** or **2** present inside (serosal fluid) and outside (mucosal fluid) of each sac was calculated by HPLC with diode-array detection (HPLC-DAD) analysis.

### 3.8. HPLC-MS Analysis

The obtained samples from the everted sac model experiment were centrifuged for 20 min at 13,500× *g* rpm, and the supernatant aqueous solution was filtered through a microporous membrane (0.45 μm pore size) for high-performance liquid chromatography-mass spectrometry analysis. LC-MS analysis was carried out using an HPLC Agilent Technologies 1260 Infinity II and a single quadrupole mass spectrometer. The employed analytical column was a Synergi Polar-RP C18 80 Å (250 mm × 4.6 mm with 4 μm particle size), and the separation of the analytes was obtained with a mobile phase composed of acetonitrile and water, both with 0.1% of formic acid, at a flow rate of 1 mL/min. The injection volume was 1 µL. The parameters of the ionization source were: drying gas flow, 12 L/min, nebulizer pressure, 50 psi, drying gas temperature, 350 °C, and capillary voltage, 4000 V. The acquisition was performed in Selected Ion Monitoring (SIM) mode in positive polarity, and the quantitative ions for compounds **1** and **2** were 247.2 and 377.2 *m*/*z*, respectively.

### 3.9. Calculation of the Apparent Permeability Coefficients

Permeability coefficients (*P_app_*) [44] were calculated according to the following equation:

*P_app_* (expressed in cm/s) is the apparent permeability coefficient, and *dQ*/*dt* (μg/s) is the analyte quantity transported across the membrane per unit of time. *A* (3.93 ± 0.038 cm^2^) is the surface area of everted gut sac intestinal mucosa available for permeation, and *C_t_* (μg/mL) is the initial analyte concentration outside the sacs. *P_app_* values were expressed in 10^−6^ cm/s units.

### 3.10. Percentage of Drug Absorption (A%) and Drug Retention (Ad%)

The concentration of analyte absorbed was calculated according to the following equation:

$$A\%=\frac{C_{s}}{C_{i}}\times 100$$

C_s_ (μg/mL) represents the analyte concentrations inside the sacs (serosal), while C_i_ (μg/mL) symbolizes the initial concentration of the drug outside (mucosal) of the everted gut sacs.

The percentage of drug retained was calculated using the equation:

$$Ad\%=100-\frac{C_{s}+C_{m}}{C_{i}}\times 100$$

C_s_ and C_m_ (μg/mL) represent the analyte concentrations quantified inside (serosal) and outside (mucosal) the sacs, respectively; C_i_ (μg/mL) is the initial concentration of the drug outside the everted gut sacs.

### 3.11. Statistical Analysis

Quantitative data are presented as means ± SE from three-five independent experiments. Biological data were analyzed using GraphPad Prism8 Software (San Diego, CA, USA), and the statistical significance of differences was assessed using a two-tailed Student’s t-test or one-way ANOVA. *p* ≤ 0.05 was taken as statistically relevant.

## 4. Conclusions

Numerous studies performed within the last two decades provide strong evidence demonstrating that CK1 isoform δ is a key player in several cellular signal transduction pathways. In addition, the over-function of A_2A_ARs was also associated with aberrant synaptic plasticity and synaptotoxicity with the onset of brain disease symptoms. Compounds **1** and **2** behaved as dual anta-inhibitors of CK1δ and A_2A_ARs, and this peculiar activity makes them potential drugs for numerous pathologies in which these two targets are involved. The results demonstrated that these compounds can counteract or restore the damage produced by the CK cocktail. In addition, compounds **1** and **2** possess antioxidant activity, which is particularly marked in compound **2**. Most kinase inhibitors are not able to cross the blood–brain barrier and are, therefore, only suitable for non-central nervous disorders. The experiments performed on everted gut sacs demonstrated that these double anta-inhibitors are able to cross the intestinal barrier and probably also to cross the blood–brain barrier, proving to be the best potential drugs for neurodegenerative diseases.

## Figures and Tables

**Table 1:** (**A**) Binding data of understudy compounds at human ARs stably transfected in CHO cells (K_i_, μM). (**B**) Enzymatic inhibition activity at 40 and 10 μM (IC_50_, μM).

	A			B		
Cmp	K_i_A_1_ (μM) ^a^	K_i_A_2A_ (μM) ^b^	K_i_A_3_ (μM) ^c^	% Residual Activity at 40 μM ± sd	% Residual Activity at 10 μM ± sd	IC_50_ (μM)
**1**	3.339 ± 0.658	0.123 ± 0.002	2.878 ± 0.695	19 ± 9.4	27 ± 4.0	1.75 ± 0.2
**2**	2.903 ± 0.648	0.0762 ± 0.173	1.146 ± 0.107	22 ± 1.0	34 ± 5.0	0.59 ± 0.2
**3**	0.118 ± 0.010	0.007 ± 0.00001	0.140 ± 0.035	86 ± 1.6	n.d.	>40
**4**	8.657 ± 1.524	3.478 ± 0.312	5.957 ± 1.021	25 ± 9.6	13 ± 24	0.36 ± 0.1
ZM **241385**	348 ± 11	1.250 ± 0.21	1072 ± 207	-	-	-
PF-**670462**	-	-	-	-	-	0.014 [33]

^a^ Displacement of specific [^3^H]CCPA binding in membranes from CHO cells stably transfected with human recombinant A_1_AR. ^b^ Displacement of specific [^3^H]NECA binding in membranes from CHO cells stably transfected with human recombinant A_2A_AR. ^c^ Displacement of specific [^3^H]HEMADO binding in membranes from CHO cells stably transfected with human recombinant A_3_AR. Data (*n* = 3–5) are expressed as means ± standard errors.

**Table 2:** Concentrations of compound **1** expressed as μg/mL absorbed in the duodenum after 60 min of incubation.

Initial Concentration	Absorbed Concentration
24.6	11.5
2.46	1.03
0.246	0.059

**Table 3:** Concentrations of compound **2** expressed as μg/mL absorbed in the duodenum after 60 min of incubation.

Initial Concentration	Absorbed Concentration
37.6	8.66
3.76	0.576
0.376	-

**Table 4:** Compound **1**. Apparent permeability coefficient P*_app_* (cm/s), percentages of drug absorption, and retention across the everted gut sac.

Concentration	Percentage of Drug Absorption (*A*%)	Percentage of Drug Retention (*Ad*%)	P*_app_* (×10^−6^ cm/s)
10^−4^ M	47	2	33
10^−5^ M	42	10	29.6
10^−6^ M	24	35	16.9

**Table 5:** Compound **2**. Apparent permeability coefficient P*_app_* (cm/s), percentages of drug absorption, and retention across the everted gut sac.

Concentration	Percentage of Drug Absorption (*A*%)	Percentage of Drug Retention (*Ad*%)	P*_app_* (×10^−6^ cm/s)
10^−4^ M	23	55	16.1
10^−5^ M	15	66	10.8
10^−6^ M	-	-	-

## Data Availability

Date sharing is contained in this article.

## Sample Availability

Samples of compounds **1** and **2** are available from the authors.
